# Policies to improve end-of-life decisions in Flemish hospitals: communication, training of health care providers and use of quality assessments

**DOI:** 10.1186/1472-684X-8-20

**Published:** 2009-12-30

**Authors:** Ina D'Haene, Robert H Vander Stichele, H Roeline W Pasman, Nele Van den Noortgate, Johan Bilsen, Freddy Mortier, Luc Deliens

**Affiliations:** 1 Heymans Institute of Pharmacology, Ghent University, Ghent, Belgium; 2 End-of-Life Care Research Group, Vrije Universiteit Brussel, Brussels, Belgium; 3 Department of Public and Occupational Health, EMGO Institute for Health and Care Research, VU University Medical Center, Amsterdam, The Netherlands; 4 Department of Geriatrics, Ghent University Hospital, Ghent, Belgium; 5 Bioethics Institute Ghent, Ghent University, Ghent, Belgium

## Abstract

**Background:**

The prevalence and implementation of institutional end-of-life policies has been comprehensively studied in Flanders, Belgium, a country where euthanasia was legalised in 2002. Developing end-of-life policies in hospitals is a first step towards improving the quality of medical decision-making at the end-of-life. Implementation of policies through quality assessments, communication and the training and education of health care providers is equally important in improving actual end-of-life practice. The aim of the present study is to report on the existence and nature of end-of-life policy implementation activities in Flemish acute hospitals.

**Methods:**

A cross-sectional mail survey was sent to all acute hospitals (67 main campuses) in Flanders (Belgium). The questionnaire asked about hospital characteristics, the prevalence of policies on five types of end-of-life decisions: euthanasia, palliative sedation, alleviation of symptoms with possible life-shortening effect, do-not-resuscitate decision, and withdrawing or withholding of treatment, the internal and external communication of these policies, training and education on aspects of end-of-life care, and quality assessments of end-of-life care on patient and family level.

**Results:**

The response rate was 55%. Results show that in 2007 written policies on most types of end-of-life decisions were widespread in acute hospitals (euthanasia: 97%, do-not-resuscitate decisions: 98%, palliative sedation: 79%). While standard communication of these policies to health care providers was between 71% and 91%, it was much lower to patients and/or family (between 17% and 50%). More than 60% of institutions trained and educated their caregivers in different aspects on end-of-life care. Assessment of the quality of these different aspects at patient and family level occurred in 25% to 61% of these hospitals.

**Conclusions:**

Most Flemish acute hospitals have developed a policy on end-of-life practices. However, communication, training and the education of health care providers about these policies is not always provided, and quality assessment tools are used in less than half of the hospitals.

## Background

In developed countries, almost one in two of all deaths occurs in a hospital. Only in recent decades have hospitals in the Western world begun to develop formal end-of-life care policies (written position papers or specific guidelines) on the appropriateness of treatments in terminal care and on end-of-life decisions, such as non-treatment decisions, palliative sedation and euthanasia. In countries such as the Netherlands and Belgium, where specific end-of-life care legislation, such as the law on patient rights [1], palliative care [2], and the law allowing euthanasia under specific circumstances [3], has been established, health care institutions of different stances felt the need to develop policies to increase transparency, accountability, consistency and quality of terminal care [4,5]. Furthermore, the enactment of the Belgian euthanasia law was followed by an increase in most types of medical end-of-life decisions. In 2007, 1.9% of all deaths in Flanders (the Dutch-speaking part of Belgium) were the result of euthanasia, 26.7% of intensified alleviation of pain, 17.4% of withholding or withdrawing life-prolonging treatment and 14.5% of palliative sedation [6].

Several studies have been conducted on the prevalence and development of policies and guidelines concerning specific end-of-life decisions used in specific settings [7-12]. In the Netherlands and in Belgium, where euthanasia was legalised in 2002, more intense and comprehensive research has been performed. In the Netherlands, research on the prevalence and implementation of policies on all end-of-life decisions in different settings providing intramural care was begun as early as 1994 [5,13-16], and repeated in 2005 [17]. In general, there were more policies and guidelines on end-of-life decisions in 2005 than in 1994 [17]. In Belgium, in 2002 the prevalence of DNR policies was studied in acute geriatric wards [18]. In 2002, Catholic hospitals and nursing homes were surveyed about the prevalence and implementation of policies on euthanasia and other end-of-life decisions. In 2005 this survey was repeated in all hospitals and nursing homes [19-21]. At the time of the survey 63% of the hospitals had a policy on euthanasia, 62% on withholding and/or withdrawing life-sustaining treatment, 27% on palliative sedation, and 14% on pain and symptom control [20] but it was unclear whether these figures were stable or growing.

The aim of the study is to describe the evolution of the penetration of formal end-of-life policies in Flemish acute hospitals and to describe implementation strategies through communication, training and education of health care providers, and quality assessments. To study actual implementation efforts of institutional policies regarding end-of-life care in one of the few countries in the world where euthanasia has been legalised is relevant as to appreciate the impact of legal change on the quality of end-of-life care.

This study will to this end address the following research questions:

▪ What is the prevalence of end-of-life policies in Flemish hospitals five years after the approval of the euthanasia law, the law on patient rights and on palliative care?

▪ How are end-of-life policies communicated internally and externally by Flemish hospitals?

▪ What is the availability of training and education in end-of-life practices in Flemish hospitals?

▪ How do Flemish hospitals assess the quality of end-of-life practices offered to patients and families? Are validated and tested measurement tools being used?

## Methods

### Design

A cross-sectional mail survey was conducted from May 2007 to October 2007 in all acute hospitals (67 main campuses) in Flanders (the Dutch speaking part of Belgium). Contact details of the hospitals were obtained from The Federal Public Service Health, Food Chain Safety and Environment. The postal questionnaires were addressed to the management of each institution, with a request to dispatch them to the person judged most suited to provide the answers. A reminder letter was sent to all non-responders two weeks after the first mailing, followed by repeated telephone follow-up after two weeks. The Ethical Review Board of Ghent University Hospital approved the study protocol.

### Definitions

A policy can consist of two distinct elements: a written position paper (opposing or allowing a specific end-of-life decision) and/or a guideline (a written protocol to guide the caregivers in approaching a problem that includes a decision-making process in a phased plan).

### Questionnaire

The questionnaire was based on similar studies in the Netherlands in 1994 [5] and 2005 [17]. We used the same questionnaire, but added questions about the availability of training and education, and activities of quality assessment regarding end-of-life care. The questionnaire consisted of 44 questions, divided into four major parts. The first part contained questions about hospital characteristics. The second part surveyed the prevalence of respectively written position papers and/or guidelines on five types of end-of-life decisions (euthanasia, palliative sedation, alleviation of symptoms with possible life-shortening effect, do-not-resuscitate decision, and withdrawing or withholding treatment). The third contained questions about internal and external communication strategies pertaining to the policy (using a three point scale: standard communication, communication on request, no communication), and the availability of training and education (internal and outsourced) (using a three point scale: yes, no but we plan to in the near future, no we don't plan to in the near future). The fourth part probed the activities of quality assessment regarding end-of-life care (using a three point scale: yes, no but we plan to in the near future, no we don't plan to in the near future), based on six categories of patient and family outcomes (physical comfort; well-being; access to information and control over treatment decisions; family spiritual, psychological and social well-being; continuity of care across providers and care settings; and family adjustment after death) [22]. We also asked whether specific measurement tools for palliative care, for which a Dutch version is available, were used, such as the Resident Assessment Instrument Minimum Data Set (RAI-MDS), the Resident Assessment Instrument for Palliative Care (RAI-PC), the Palliative care Outcome Scale (POS), and the Edmonton Symptom Assessment System (ESAS), whether the hospital applied a structured approach to palliative care (defined as the planning of medical and care procedures, including diagnostic tests, medication and consultations intended to develop an efficient and coordinated treatment programme), and finally whether the end-of-life care policy was integrated into the quality handbook of the institution.

The questionnaire was tested for suitability in the Flemish context with five professionals working in acute hospitals (two general directors, one medical director, a member of the board of directors and a geriatrician). Their comments resulted in a limited number of adaptations.

### Statistical analysis

Data analyses were performed with descriptive statistics, SPSS software (release 15.0).

## Results

### Sample description and representativity (Table 1)

**Table 1:** Characteristics of the response sample compared to the overall population of Flemish hospitals in 2007^1^

Characteristic	Responding hospitals (N = 37)	Population^2 ^(N = 67)
**Size of hospitals (bed capacity)**	N (%)	N (%)
<250 beds	6 (16)	17 (25)
250-500 beds	14 (38)	27 (40)
501-750 beds	9 (24)	13 (19)
>750 beds	8 (22)	10 (15)

**Palliative care unit**	N (%)	N (%)
Yes	17 (46)	27 (40)

**Membership in umbrella organisation**	N (%)	N (%)
VVI^3^	21 (57)	41 (61)
VOV^4^	11 (30)	21 (31)
None	5 (14)	5 (8)

**Religious affiliation**	N (%)	N (%)
Catholic	22 (59)	42 (63)

^1 ^Absolute numbers (with rounded percentages in parenthesis)

^2 ^Data provided by The Federal Public Service Health, Food Chain Safety and Environment

^3 ^VVI, Verbond der Verzorgingsinstellingen: umbrella organisation that assembles Catholic healthcare institutions in Flanders

^4 ^VOV, Vereniging van Openbare Verzorgingsinstellingen: umbrella organisation that assembles public hospitals in Flanders

Of the 67 acute hospitals (main campuses) in Flanders, 37 completed and returned the questionnaire, which represents a 55% response rate.

Thirty-eight percent of the hospitals had more than 500 beds. Fifty-nine percent of the participating hospitals had a religious affiliation, and 46% had a palliative care unit. All hospitals had an ethics committee as required by Belgian law.

Characteristics of responding hospitals were similar to the population for size, availability of a palliative care unit, membership of an umbrella organisation and religious affiliation. All differences were statistically non-significant and smaller than 10 percentage points (Table 1).

The management of the hospital receiving the questionnaire dispatched it for completion to: a member of the ethics committee (38%), the medical director (14%), a member of the palliative support team (14%) or the general director (8%). The rest of the questionnaires were completed by another person (e.g. nursing director) or by more than one person (e.g. the medical director and a member of the ethics committee) (data not shown).

### Prevalence of end-of-life policies (Figure 1)

**Figure 1:**
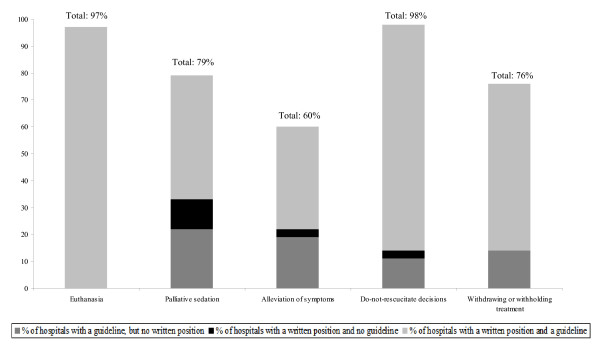
**Prevalence (in percentage) of policy documents on all end-of-life decisions in Flemish hospitals in 2007 (N = 37)^1^**. ^1 ^Missing values: Palliative sedation: 2; Alleviation of symptoms: 5; Withdrawing or withholding treatment: 1.

Figure 1 shows the prevalence of policies for all end-of-life decisions. The prevalence of policies on euthanasia and do-not-resuscitate decisions was over 97% in Flemish hospitals. All but one of the hospitals (97%) in the response sample had a written position and a practical guideline on euthanasia. A high number of hospitals had a written position, a guideline, or both with regard to palliative sedation (79%) and withdrawing or withholding treatment (76%). The lowest prevalence was found for policies on alleviation of symptoms (60%).

The content of the written policies on euthanasia varies (data not shown). Ten of the surveyed hospitals (28%) leave the decision to apply euthanasia entirely to physicians. One third allows euthanasia in accordance with the law, and another third of the hospitals allows euthanasia provided additional conditions are applied (e.g. additional palliative care procedures and/or individual approval by an ethics committee). No hospitals reported not allowing euthanasia.

### Communication of written positions on end-of-life decisions (Table 2)

**Table 2:** Communication of written positions on all end-of-life decisions in Flemish hospitals in 2007^1-2^

	Standard communication	Communication on request	No communication
**Euthanasia (n = 36)**	N (%)	N (%)	N (%)
Physicians	26 (76)	4 (12)	4 (12)
Nursing staff	28 (82)	4 (12)	2 (6)
External referrals	13 (45)	10 (35)	6 (21)
Patients and/or family	5 (17)	16 (53)	9 (30)

**Palliative sedation (n = 22)**	N (%)	N (%)	N (%)
Physicians & nursing staff	15 (71)	5 (24)	1 (5)
Patients and/or family	5 (29)	12 (71)	0

**Alleviation of symptoms (n = 15)**	N (%)	N (%)	N (%)
Physicians & nursing staff	13 (87)	2 (13)	0
Patients and/or family	5 (38)	8 (62)	0

**Do-not-resuscitate decisions (n = 32)**	N (%)	N (%)	N (%)
Physicians & nursing staff	27 (87)	4 (13)	0
Patients and/or family	13 (50)	12 (46)	1 (4)

**Withdrawing or withholding treatment (n = 23)**	N (%)	N (%)	N (%)
Physicians & nursing staff	21 (91)	2 (9)	0
Patients and/or family	9 (45)	11 (55)	0

^1 ^Absolute numbers (with rounded percentages in parenthesis)

^2 ^Between 1 and 7 missing values

Communicating written positions on euthanasia (N = 36) to physicians, nurses and external referring physicians, was standard practice in respectively 76%, 82% and 45% of the hospitals. Patients, however, mostly had to place a request in order to receive a copy (53%).

The communicating to physicians and nursing staff of the written positions on do-not-resuscitate decisions (N = 32), palliative sedation (N = 22) and alleviation of symptoms (N = 15) was the rule in 87%, 71% and 87% of the hospitals respectively while patients and families receive them on request in 46%, 71% and 62% of the hospitals respectively (Table 2).

### Availability of end-of-life care training and education (Table 3)

**Table 3:** Availability of training and education and other initiatives in end-of life care in Flemish hospitals in 2007 (N = 37)^1-2^

	Yes	No, but plan to	No, don't plan to
**Availability of training and education of health care providers in end-of-life care**	N (%)	N (%)	N (%)
Pain & symptom control	36 (97)	1 (3)	0
Spiritual, psychological & social care for patients	33 (89)	4 (11)	0
Law & regulation	30 (88)	3 (9)	1 (3)
Training in the use of own guidelines	29 (83)	3 (9)	3 (9)
Spiritual, psychological & social care for relatives	28 (82)	4 (12)	2 (6)
Bereavement care	24 (69)	8 (23)	3 (9)
Communication skills with regard to treatment possibilities & care planning	24 (67)	8 (22)	4 (11)

**Availability of other initiatives in end-of-life care**	N (%)	N (%)	N (%)
Integration of the end-of-life care policy into the quality handbook	16 (43)	15 (41)	6 (16)
Structured approach to palliative care	12 (33)	15 (42)	9 (25)

^1 ^Absolute numbers (with rounded percentages in parenthesis)

^2 ^Between 1 and 3 missing values

Training and education in pain and symptom control and in spiritual, psychological and social care for patients was widely available to physicians and health care providers in acute hospitals (97% and 89% respectively). The lowest percentages were found for communication skills with regard to treatment possibilities and care planning (67%), and for bereavement care (69%). However, almost a quarter of hospitals reported an intention to plan training and education on these topics in the near future (Table 3).

Concerning a structured approach to palliative care, 42% of the hospitals surveyed planned, and 33% had already implemented such an approach. Almost half of the hospitals (43%) mentioned their end-of-life care policy in the quality handbook and 41% planned to integrate the end-of-life care policy in the near future.

### Prevalence of quality assessment in end-of-life care (Table 4)

**Table 4:** Prevalence of quality assessment in end-of-life care in Flemish hospitals in 2007 (N = 37)^1-2^

	Yes	No, but plan to	No, don't plan to
**Assessment of patient satisfaction with**	N (%)	N (%)	N (%)
pain & symptom control	22 (61)	7 (19)	7 (19)
spiritual, psychological & social care	17 (49)	9 (26)	9 (26)

**Assessment of relatives satisfaction with**	N (%)	N (%)	N (%)
spiritual, psychological & social care	11 (33)	9 (27)	13 (39)
continuity of care (between ≠ care settings & caregivers)	11 (33)	8 (24)	14 (42)
bereavement care	8 (25)	7 (21)	18 (55)

^1 ^Absolute numbers (with rounded percentages in parenthesis)

^2 ^Between 1 and 4 missing values

Sixty-one percent of the hospitals assess the satisfaction of patients with pain and symptom control, whereas 49% assess patient satisfaction with spiritual, psychological and social care, 33% relatives' satisfaction with spiritual, psychological and social care, 33% continuity of care, and 25% bereavement care. Between 19% and 55% of the hospitals reported explicitly that they do not plan such assessments in the future.

The use of internationally validated and tested measurement tools for palliative care (RAI-MDS, RAI-PC, POS, ESAS) was very low to non-existent. Of all hospitals (n = 37) only four (11%) reported to use the ESAS, one (3%) the POS, and none the RAI-MDS or RAI-PC. Six hospitals (16%) reported to use other tools, such as the DOLOPLUS, the STAS and the Distress Barometer (data not shown).

## Discussion

This is the first study to probe not only the prevalence and implementation of policies in end-of-life care in acute hospitals in Flanders but also to assess efforts to implement them through communication, training and educating health care providers, and quality assessments on a patient and family level. Results show that in 2007 policies on most types of end-of-life decisions (euthanasia, do-not-resuscitate decisions, palliative sedation) were widespread in acute hospitals. Efforts were being undertaken to communicate these policies internally in hospitals and to a somewhat lesser extent externally as well. Dependent on the content (65% to 97%), large numbers of acute hospitals train and educate their health care providers in different aspect of end-of-life care, and in the use of their own end-of-life care policies. Generally the assessment of the quality of different aspects of end-of-life care is not yet common, except for the measurement of satisfaction with pain and symptom control which is done by 61% of the hospitals.

There are several limitations to the study. The response rate is marginally acceptable (55%) and could be problematic in a sensitive survey on ethical matters in health care institutions with different life stances. We sent the survey to the management of hospitals, with a request to dispatch it to the most appropriate person. This two-step approach may have hampered the response rate by channelling the questionnaire to persons undertaking a variety of functions. In addition, these different types of respondents may have had different familiarity with hospital policy. Nevertheless, we observed no statistical and relevant differences between sample and population, including religious affiliation, as far as the surveyed characteristics are concerned.

We also acknowledge that the results, as they are based on self-reported data, are potentially subject to both recall bias and social desirability bias. Therefore answers may not adequately reflect actual practices within institutions.

A final limitation we would like to mention is that the number of hospitals in Flanders is too small to permit a detailed and adequately powered analysis of institutional characteristics.

It is well established that the approval of the euthanasia law in Belgium in 2002 (and of the law on patient rights and on palliative care enacted in the same year) resulted in an intensified debate on how to deal with euthanasia requests within Belgian hospitals [20]. This presumably contributed to a prevalence of written policies on euthanasia of approximately 60% in hospitals in 2005 [20]. Meanwhile our results show an almost universal implementation of policies on euthanasia in acute hospitals five years after the approval of the law. However, our data do not permit us to distinguish between hospitals which issue guidelines in order to enhance the quality of care of decision-making and those which do so to impose additional hurdles with the intention of rendering the practice of euthanasia more difficult.

The prevalence of other policies on end-of-life care is also high in Flemish acute hospitals. Policies on do-not-resuscitate decisions are almost universally implemented, following a trend started in 1985 (with a step-up in 1997 and 2001) in acute geriatric wards [18]. There has also been an increase in the number of do-not-resuscitate decisions since 1994, possibly influenced by the increase in do-not-resuscitate policies [18]. The prevalence of palliative sedation policies jumped from 27% in 2005 [20] to 79% in 2007, according to this study, which is remarkable, because this practice was virtually unknown before 2001 [23]. Similarly, the prevalence of policies on non-treatment decisions rose from 14% to 76%, indicating more attention to and regulation of these practices, although they are considered as regular medical practices and are not affected by the euthanasia law. The prevalence of policies on alleviation of symptoms, by contrast, remained stable at 60%. Our study shows that in 2007, in most Flemish acute hospitals, specific policies are available covering a wide array of end-of-life decisions, including euthanasia. Prevalence percentages of end-of-life policies in Flemish hospitals are high compared to the results of the Dutch study in 2005 where 89% of the hospitals had practice guidelines for euthanasia, 41% for palliative sedation, 39% for intensified alleviation of symptoms with possible life-shortening effect, and 48% for withholding or withdrawing treatment [17]. However, a further rise in prevalence of policies in Dutch hospitals in the past years is probable.

Taking the potential benefits of policies into account, such as supporting quality improvement initiatives, and enhancing accountability and transparency [4,5], these instruments can be regarded as an important step towards improving the quality of medical decision-making at the end-of-life. Our study shows that a considerable amount of effort is made in Flemish hospitals to implement existing policies on end-of-life care, though not, however, by all hospitals. Many hospitals do indeed invest in communicating their written positions and in providing training and education on their policies to physicians and health care providers, which raises awareness of these instruments [24]. Nevertheless, about 10% of the hospitals surveyed in our study reported that they do not plan to undertake formal training in the use of their own guidelines. It is also clear that in the communication of written positions to patients and/or families, a reactive approach is preferred, limited to personal conversations when patients or family request information. These documents are thus rarely used as a tool to improve shared decision-making and communication regarding end-of-life care [18].

It is encouraging to see that many hospitals offer supportive services such as spiritual, psychological and social end-of-life care to patients, invest in the training and education of their physicians and health care providers in several end-of-life care areas and apply or plan to apply a structured care approach to palliative care, and the integration of the end-of-life care vision into the quality handbook.

These results describe an important development in the monitoring of the quality of end-of-life practices in general hospitals in Flanders. Most hospitals have implemented policies and are at the crucial stage of implementing these actively. However, the next step i.e. the introduction of a method of assessment of quality of end-of-life practices and of patients' needs seems not to be so obvious to them. The number of hospitals that currently measure or plan to measure satisfaction on the patient and family level is low, except for satisfaction with pain and symptom control on the patient level. A considerable number of hospitals even reported that they do not intend to develop such strategies in the future. Furthermore, internationally validated and tested measurement tools such as the Palliative care Outcome Scale are seldom used, as has been found by other researchers [22]. It is however essential to determine whether activities designed to improve the quality of end-of-life care are actually having an impact. The implementation of quality assessment strategies for this purpose is of vital importance in gaining insight into the quality of the end-of-life care offered to patients and family. Based on these assessments, challenges and opportunities in end-of-life care can be identified and possibly tackled.

## Conclusions

Our study shows a growing awareness of the importance of end-of-life policies and the implementation of these policies in acute hospitals in Flanders. A continued investment of resources and cooperation between researchers, health care providers, national organizations, and governmental agencies will be needed to sustain implementation strategies, and to create a measurement-driven approach to quality assurance in end-of-life care.

## Competing interests

The authors declare that they have no competing interests.

## Authors' contributions

All authors contributed to the manuscript. The manuscript was drafted by ID, with further input from all other authors. LD, RVS and HRWP are the project supervisors. All authors read, revised and approved the final manuscript.

## Pre-publication history

The pre-publication history for this paper can be accessed here:

http://www.biomedcentral.com/1472-684X/8/20/prepub

## References

[B1] Belgisch Staatsblad 26 september 2002 [Belgian official collection of the laws September 26 2002]: Wet betreffende de rechten van de patient 22 augustus 2002 [Law concerning patient rights in Belgium August 22, 2002] (in Dutch) Number Bill 2002022737 Brussels, Belgium 2002

[B2] Belgisch Staatsblad 26 oktober 2002 [Belgian official collection of the laws Octobre 26 2002]. Wet betreffende palliative zorg 14 juni 2002 [Law concerning palliative care Belgium June 14, 2002] (in Dutch) Number Bill 2002022868 Brussels, Belgium 2002

[B3] Belgisch Staatsblad 22 juni 2002 [Belgian official collection of the laws June 22 2002]. Wet betreffende euthanasie 28 mei 2002 [Law concerning euthanasia May 28, 2002] (in Dutch) Number Bill 2002009590 Brussels, Belgium 2002

[B4] Woolf SH Grol R Hutchinson A Eccles M Grimshaw J Clinical guidelines: potential benefits, limitations, and harms of clinical guidelines BMJ 1999 318 527 530 10024268 10.1136/bmj.318.7182.527 PMC1114973

[B5] Haverkate I van Delden JJ van Nijen AB Wal G van der Guidelines for the use of do-not-resuscitate orders in Dutch hospitals Crit Care Med 2000 28 3039 3043 10.1097/00003246-200008000-00060 10966292

[B6] Bilsen J Cohen J Chambaere K Pousset G Onwuteaka-Philipsen BD Mortier F Deliens L Medical end-of-life practices under the euthanasia law in Belgium N Engl J Med 2009 361 1119 1121 10.1056/NEJMc0904292 19741238

[B7] Rasooly I Lavery JV Urowitz S Choudhry S Seeman N Meslin EM Lowy FH Singer PA Hospital policies on life-sustaining treatments and advance directives in Canada CMAJ 1994 150 1265 1270 8162549 PMC1486449

[B8] Caplan AL Snyder L Faber-Langendoen K The role of guidelines in the practice of physician-assisted suicide. University of Pennsylvania Center for Bioethics Assisted Suicide Consensus Panel Ann Intern Med 2000 132 476 481 10733448 10.7326/0003-4819-132-6-200003210-00009

[B9] Cook DJ Guyatt G Rocker G Sjokvist P Weaver B Dodek P Marshall J Leasa D Levy M Varon J Fisher M Cook R Cardiopulmonary resuscitation directives on admission to intensive-care unit: an international observational study Lancet 2001 358 1941 1945 10.1016/S0140-6736(01)06960-4 11747918

[B10] Braun TC Hagen NA Clark T Development of a clinical practice guideline for palliative sedation J Palliat Med 2003 6 345 350 10.1089/109662103322144655 14509479

[B11] Clarke EB Luce JM Curtis JR Danis M Levy M Nelson J Solomon M A content analysis of forms, guidelines, and other materials documenting end-of-life care in intensive care units J Crit Care 2004 19 108 117 10.1016/j.jcrc.2004.05.001 15236144

[B12] Morita T Bito S Kurihara Y Uchitomi Y Development of a clinical guideline for palliative sedation therapy using the Delphi method J Palliat Med 2005 8 716 729 10.1089/jpm.2005.8.716 16128645

[B13] Haverkate I Muller MT Cappetti M Jonkers FJ Wal G van der Prevalence and content analysis of guidelines on handling requests for euthanasia or assisted suicide in Dutch nursing homes Arch Intern Med 2000 160 317 322 10.1001/archinte.160.3.317 10668833

[B14] Haverkate I Wal G van der Dutch nursing home policies and guidelines on physician-assisted death and decisions to forego treatment Public Health 1998 112 419 423 10.1016/S0033-3506(98)00290-X 9883041

[B15] Haverkate I Wal G van der Policies on assisted suicide in Dutch psychiatric facilities Psychiatr Serv 1998 49 98 100 9444688 10.1176/ps.49.1.98

[B16] Haverkate I Wal G van der Policies on medical decisions concerning the end of life in Dutch health care institutions JAMA 1996 275 435 439 10.1001/jama.275.6.435 8627954

[B17] Pasman HRW Hanssen-de Wolf JE Hesselink BAM Heide A van der Wal G van der Maas PJ van der Onwuteaka-Philipsen BD Policy statements and practice guidelines for medical end-of-life decisions in Dutch health care institutions: developments in the past decade Health Policy 2009 92 1 79 88 10.1016/j.healthpol.2009.02.008 19342116

[B18] De Gendt C Bilsen J Stichele R Vander Lambert M Noortgate N Van Den Deliens L Do-not-resuscitate policy on acute geriatric wards in Flanders, Belgium J Am Geriatr Soc 2005 53 2221 2226 10.1111/j.1532-5415.2005.00503.x 16398913

[B19] Gastmans C Lemiengre J Wal G van der Schotsmans P Dierckx de Casterlé B Prevalence and content of written ethics policies on euthanasia in Catholic healthcare institutions in Belgium (Flanders) Health Policy 2006 76 169 178 10.1016/j.healthpol.2005.09.003 16221504

[B20] Lemiengre J Dierckx de Casterlé B Verbeke G Guisson C Schotsmans P Gastmans C Ethics policies on euthanasia in hospitals--A survey in Flanders (Belgium) Health Policy 2007 84 170 180 10.1016/j.healthpol.2007.05.007 17618011

[B21] Lemiengre J Dierckx de Casterlé B Verbeke G Van Craen K Schotsmans P Gastmans C Ethics policies on euthanasia in nursing homes: a survey in Flanders, Belgium Soc Sci Med 2008 66 376 386 10.1016/j.socscimed.2007.09.007 17996349

[B22] Casarett DJ Teno J Higginson I How should nations measure the quality of end-of-life care for older adults? Recommendations for an international minimum data set J Am Geriatr Soc 2006 54 1765 1771 10.1111/j.1532-5415.2006.00925.x 17087706

[B23] Bilsen J Stichele R Vander Broeckaert B Mortier F Deliens L Changes in medical end-of-life practices during the legalization process of euthanasia in Belgium Soc Sci Med 2007 65 803 808 10.1016/j.socscimed.2007.04.016 17490798

[B24] Sinuff T Kahnamoui K Cook DJ Giacomini M Practice guidelines as multipurpose tools: a qualitative study of noninvasive ventilation Crit Care Med 2007 35 776 782 10.1097/01.CCM.0000256848.47911.77 17235258

